# Novel Genetic Variants Expand the Functional, Molecular, and Pathological Diversity of *KCNA1* Channelopathy

**DOI:** 10.3390/ijms24108826

**Published:** 2023-05-16

**Authors:** Kelsey Paulhus, Edward Glasscock

**Affiliations:** Department of Biological Sciences, Southern Methodist University, Dallas, TX 75275, USA; kpaulhus@smu.edu

**Keywords:** *KCNA1*, Kv1.1, epilepsy, episodic ataxia, myokymia, SUDEP, respiration, genetic modifiers, musculoskeletal, nystagmus

## Abstract

The *KCNA1* gene encodes Kv1.1 voltage-gated potassium channel α subunits, which are crucial for maintaining healthy neuronal firing and preventing hyperexcitability. Mutations in the *KCNA1* gene can cause several neurological diseases and symptoms, such as episodic ataxia type 1 (EA1) and epilepsy, which may occur alone or in combination, making it challenging to establish simple genotype–phenotype correlations. Previous analyses of human *KCNA1* variants have shown that epilepsy-linked mutations tend to cluster in regions critical for the channel’s pore, whereas EA1-associated mutations are evenly distributed across the length of the protein. In this review, we examine 17 recently discovered pathogenic or likely pathogenic *KCNA1* variants to gain new insights into the molecular genetic basis of *KCNA1* channelopathy. We provide the first systematic breakdown of disease rates for *KCNA1* variants in different protein domains, uncovering potential location biases that influence genotype–phenotype correlations. Our examination of the new mutations strengthens the proposed link between the pore region and epilepsy and reveals new connections between epilepsy-related variants, genetic modifiers, and respiratory dysfunction. Additionally, the new variants include the first two gain-of-function mutations ever discovered for *KCNA1*, the first frameshift mutation, and the first mutations located in the cytoplasmic N-terminal domain, broadening the functional and molecular scope of *KCNA1* channelopathy. Moreover, the recently identified variants highlight emerging links between *KCNA1* and musculoskeletal abnormalities and nystagmus, conditions not typically associated with *KCNA1*. These findings improve our understanding of *KCNA1* channelopathy and promise to enhance personalized diagnosis and treatment for individuals with *KCNA1*-linked disorders.

## 1. Introduction

The *KCNA1* gene encodes Kv1.1 voltage-gated potassium channel α subunits and has been linked to human disease since the 1990s when it was identified as the causative gene for an episodic ataxia and myokymia syndrome [1]. Since then, mutations in *KCNA1* have been associated with a wide variety of other diseases including epilepsy, hypomagnesemia, paroxysmal movement disorders, hyperthermia, and combinations of these pathologies. This broad spectrum of disease manifestations associated with *KCNA1* variants complicates simple genotype–phenotype correlations. 

In a previous review of pathogenic and likely pathogenic *KCNA1* mutations, we identified links between genotype and disease phenotype, particularly for mutations associated with epilepsy, which tend to cluster in regions critical for the function of the channel’s pore [2]. In this review, we examine 17 additional recently discovered *KCNA1* variants classified as pathogenic or likely pathogenic (bolded and underlined in Table 1). We identified these variants through a search of ClinVar, dbSNP, and PubMed databases. To expand our understanding of *KCNA1* phenotypic variability and potential location biases influencing genotype–phenotype correlations, we provide the first comprehensive breakdown of the disease rate for variants according to the protein domain. The discovery of new variants strengthens the linkage between the pore region and epilepsy and provides new insights into the relationship between epilepsy-related variants, genetic modifiers, and respiratory dysfunction. Additionally, recently identified variants reveal potential correlations between *KCNA1*-channelopathy and musculoskeletal abnormalities and nystagmus. These new findings enhance our understanding of *KCNA1* channelopathy and will advance personalized diagnosis and treatment for patients with *KCNA1*-linked disorders.

## 2. *KCNA1* Gene Structure and Function

The *KCNA1* gene encodes Kv1.1 voltage-gated potassium channel α subunits, which are one of 40 different Kv α-subunits spread across 12 different gene subfamilies (Kv1-12) [60,61,62]. Kv channels form functional pores through the assembly of four α subunits either as homo- or heterotetramers [63,64,65]. These tetramers associate with β subunits, which further regulate the structure, gating, assembly, and trafficking properties of the channels [66]. In the case of Kv1.1, it usually forms heterotetramers in vivo by combining with Kv1.2, Kv1.4, or Kv1.6 subunits [67]. Kv1.1-containing channels are crucial for preventing neuronal hyperexcitability by the regulation of action potential shape, repolarization, and firing properties [68]. 

**Figure 1:**
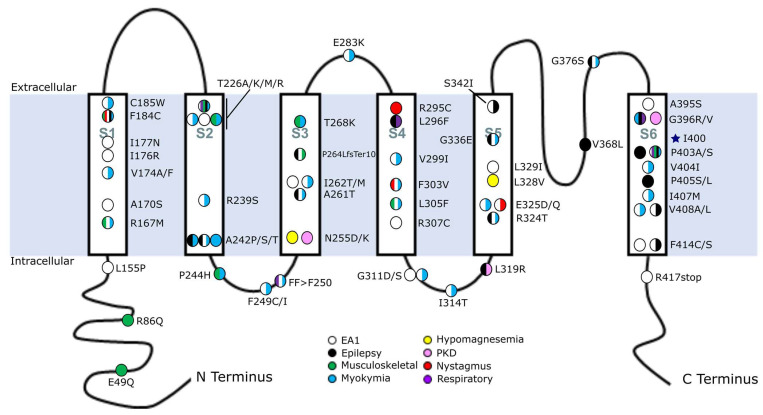
Map of *KCNA1* mutations associated with human disease. Human mutations in *KCNA1* were mapped across the protein and color-coded to indicate their clinically documented disease association. Circles with multiple colors represent mutations with multiple phenotypes. Multiple circles at a given amino acid position represent different amino acid substitutions at that location (e.g., A242P/S/T) and their associated disease manifestation. The blue star indicates the RNA editing position. The identity of the various transmembrane domains is indicated as S1–S6. Abbreviations: EA1, episodic ataxia type 1; PKD, paroxysmal kinesigenic dyskinesia.

The human Kv1.1 protein is 495 amino acids long and includes six transmembrane (TM) regions (S1–S6) that are joined by alternating extra- and intracellular linkers and flanked by intracellular N- and C-termini (Figure 1). The S1–S4 regions comprise the voltage-sensing domain of the protein, and the S5–S6 regions form the pore domain of the channel [62,69]. S4 is critical for voltage sensing, as it is made up of evenly spaced positive residues that can accurately sense fluctuations in membrane potential and interact with S3 to bring about conformational changes that alter the channel’s open state [69,70,71]. The extracellular linker between S5 and S6 acts as a K^+^ selectivity filter [72]. The roles of the N- and C-termini are not fully understood, but it is hypothesized that the N-terminus is important for channel subunit assembly, while the C-terminus is involved in tetramerization and targeting of the channel to the membrane [73,74].

## 3. Previous Genotype–Phenotype Correlations

Understanding genotype–phenotype correlations for *KCNA1* channelopathy is challenging because mutations can result in a variety of different diseases, which often occur in combination. Historically, three diseases have been predominantly associated with *KCNA1* mutations, namely episodic ataxia type 1 (EA1), myokymia, and epilepsy. Among these, the most common is EA1, a rare genetic paroxysmal movement disorder that can be triggered by stress resulting in impaired voluntary movements such as walking [14,35,60]. Out of approximately 65 known pathogenic or likely pathogenic *KCNA1* mutations, including more recent ones reviewed here, 69% cause EA1 (Table 2). Myokymia is the second most common, linked to 52% of *KCNA1* variants and usually occurring in combination with EA1. Myokymia is characterized by episodes of involuntary muscle rippling arising from abnormal peripheral nerve activity [16]. The third most common phenotype associated with *KCNA1* variants is epilepsy or seizures, accounting for approximately 32% of mutations. Clinical case reports often describe patients experiencing seizures without stating an official epilepsy diagnosis, so this category comprises patients with either documented epilepsy or seizures. Although not traditionally recognized, it is now becoming increasingly apparent that musculoskeletal abnormalities and nystagmus can also be features of *KCNA1* channelopathy, occurring in 17% and 6% of *KCNA1* mutations, respectively. Importantly, at least 60% of *KCNA1* mutations cause more than one type of disease. This high degree of comorbidities complicates simple genotype–phenotype correlations.

The location of the mutation within the protein appears to play a role in determining the type of disease that manifests. A previous review revealed that mutations causing EA1 are generally distributed throughout the whole length of the Kv1.1 protein except for the intracellular N-terminal region, which has heretofore not contained any pathogenic mutations [2]. In contrast, epilepsy- or seizure-associated variants tend to cluster in the protein’s pore domain and to a lesser degree in specific regions of S1 and S2 that are important for voltage-sensing and stabilizing the pore’s open state [2]. The epilepsy-causing mutations affecting the pore domain were found to reside in or immediately adjacent to the pore-forming TM domains S5 and S6 or in the linker region between S5 and S6 [2]. Of special interest were several mutations that sit in the critical conserved proline–valine–proline (PVP) sequence of S6 that forms the pore activation gate [75]. Mutations in this PVP motif were highly enriched for a more severe form of epilepsy called epileptic encephalopathy (EE), which also shows comorbid cognitive impairment [2,76]. 

In this review, we will describe how new pathogenic *KCNA1* mutations are expanding our understanding of genotype–phenotype correlations associated with *KCNA1* channelopathy. We examine how these variants refine our knowledge of mutations that cause epilepsy and support our previous understanding of the molecular nature of EA1. In addition, we explore emerging links between *KCNA1* and musculoskeletal abnormalities and nystagmus that are being revealed by recently described mutations. 

## 4. New Epilepsy- or Seizure-Related Variants

Out of the 17 new *KCNA1* variants examined in this review, eight are linked to epilepsy or seizures (Table 1, Figure 1). A previous study of *KCNA1* mutations discovered that most of the variants related to epilepsy or seizures occur in the protein’s pore domain, comprising the S5, S6, and S5–S6 linker domains [2]. The newly described *KCNA1* variants further support this initial genotype–phenotype association by strengthening the correlation between mutations in the pore domain and epilepsy. Moreover, these newly identified variants indicate that mutations in specific regions of the voltage-sensing domain may also contribute to epilepsy, albeit to a lesser extent.

Among the new epilepsy-linked variants are several firsts, such as the first two gain-of-function (GOF) mutations ever discovered for *KCNA1* and the first frameshift mutation. These new variants broaden the scope of known pathogenic functional and molecular changes in *KCNA1* that can cause epilepsy. Finally, the recently identified variants highlight the potential significance of genetic modifiers in influencing the disease phenotype. They also suggest a potential association between epilepsy and respiratory dysfunction in *KCNA1* channelopathy.

### 4.1. Variants in the Pore Domain

Among the eight recently identified epilepsy- or seizure-related mutations in *KCNA1*, four reside in S5–S6 (G336E, G376S, G396R, and P403A), strengthening the association between mutations in the pore domain and epilepsy or seizures [29,49,52,54]. Overall, approximately two-thirds of all known *KCNA1* mutations that cause epilepsy or seizures localize to the S5–S6 pore domain region of Kv1.1. Analysis of the percentage of total epilepsy- or seizure-related *KCNA1* mutations by protein domain reveals that variants in S5–S6 are nearly twice as likely (43–100%) to cause epilepsy as mutations anywhere else in the protein (0–38%; Table 2). 

One of the most critical regions of the pore domain for epilepsy is the PVP motif in S6. The PVP motif is critical for ion conduction as it contains the amino acids that must bend to mediate the opening of the activation gate for the passage of K^+^ ions [75]. The prolines of the PVP motif have an especially strong association with a risk of epilepsy as all four of the *KCNA1* missense mutations that affect these residues cause epilepsy [54,55,57]. The second proline (P405) appears to be particularly important as both known mutations of that residue cause a very severe form of epilepsy called epileptic encephalopathy, which is defined by early onset epilepsy that progressively impairs brain function leading to cognitive, behavioral, and language deficits [55,57,76]. The newly identified P403A variant, which was found in a female patient with early onset epilepsy and neurodevelopmental delay that evolved over time into intellectual disability [54], further bolsters the linkage between the PVP motif in S6 and epilepsy. 

### 4.2. Variants Not in the Pore Domain

Of the *KCNA1* variants occurring outside of the S5–S6 pore domain, only 18% (7/40, not counting the P264LfsTer10 variant) cause epilepsy or seizure phenotypes, and these seem to primarily affect locations that control voltage-sensing or that indirectly affect pore function by influencing the stability of the open-state configuration of the channel [2]. However, mutations in the S2 domain exhibit a significantly higher risk with 38% (3/8) of variants causing epilepsy or seizures (Table 2).

#### 4.2.1. Gain-of-Function Mutations That Alter Voltage-Sensing

Two new *KCNA1* variants were recently identified that exert gain-of-function effects: A261T and L296F [28,29,37]. These mutations are notable for being the first of their kind. Previously identified pathogenic *KCNA1* variants were classified as causing either loss-of-function of Kv1.1 or dominant negative effects on Kv1.1-containing channels. The A261T and L296F variants also build on previous observations of a potential relationship between epilepsy and select regions of the protein that govern voltage-sensing [2]. 

Two different patients were identified with A261T variants. Beginning at 1 year and 9 months of age, the first patient, a male, exhibited focal seizures that were triggered by fever and often accompanied by episodic ataxia and myokymia [29]. At 7 years old, the second patient, a female, began having fever-induced focal occipital seizures with visual hallucinations that secondarily generalized into tonic-clonic seizures, but episodic ataxia symptoms were absent [28]. A261T is notable for being the first *KCNA1* mutation found that exerts a GOF effect, causing a 20-mV hyperpolarizing shift in steady-state activation gating, which allows the channel to open at more negative membrane potentials [28]. It is also the first variant associated with seizures or epilepsy to be located in S3 [29]. The location of this amino acid in S3 corresponds to a narrow hydrophobic layer in the voltage-sensing domain, which forms a focused transmembrane electric field through which the gating charges on S4 have to travel during voltage gating [28,77]. Of note, a previously identified mutation in S1, F184C, also affects this conserved hydrophobic layer resulting in seizures [12]. In the case of the A261T mutation, it has been hypothesized that a polar T residue at this location in S3 may confer GOF effects by facilitating the outward displacement of S4 that occurs during pore opening [28]. 

The L296F mutation was identified in an early infant male patient with a highly drug-resistant form of focal onset epilepsy associated with prolonged ictal/postictal oxygen desaturation and cyanosis [37]. L296F is located in the central portion of the S4 transmembrane domain that contains the positively charged amino acids that carry the gating charge [69]. This mutation is unique because, similar to the A261T variant, it not only acts as a GOF mutation but also confers an even larger GOF effect. Functional studies show that L296F causes a very large hyperpolarizing shift in the voltage dependence of activation of ~40 mV, which is approximately double that seen for the A261T mutation [28,37]. Therapeutic administration of low doses of the potassium channel blocker 4-aminopyridine provided improved but incomplete seizure control [37]. In addition to L296F, nine other epileptic encephalopathy-associated variants have been identified in identical or nearby positions in three related Kv channel subunit genes: *KCNA2*, *KCNQ2*, and *KCNQ3* [37,78,79,80,81,82,83]. All these mutations also cause GOF phenotypes [78,79,80,81,82,83]. Thus, the L296 amino acid may represent a mutational intolerance hotspot for GOF mutations. 

The mechanism by which these GOF mutations cause epilepsy and seizures has not been determined yet, but studies of GOF variants in other K^+^ channel genes provide some potential insights. One possibility is that mutant channel subunits present in inhibitory interneurons of epileptogenic circuits may decrease their activity, leading to disinhibition and increased network hyperexcitability [84]. Alternatively, the shift in the voltage dependence of activation towards more negative membrane potentials could induce membrane hyperpolarization, facilitating the recovery of voltage-gated Na^+^ channels from inactivation and enabling neurons to fire at a higher frequency [84]. Supporting a Na^+^ channel-related mechanism, the Na^+^ channel blocker carbamazepine effectively improved seizure management in patients with the A261T mutation [28,29].

#### 4.2.2. Mutations Hypothesized to Alter Open-State Stability

An A242S variant was recently found in a male patient diagnosed with West Syndrome, EA1, and myokymia [20]. West Syndrome is a type of epileptic encephalopathy characterized by infantile spasms, developmental regression, and hypsarrhythmia patterns on electroencephalograms (EEG) [85]. The A242S mutation resides at the intracellular end of S2 only nine amino acids away from a phenylalanine (F232) that has been shown to be vital for stabilizing the pore’s open configuration [39,86,87,88]. Another variant at A242 (A242P) was previously identified in a patient with myokymia and epilepsy [6,19]. In electrophysiological studies, the A242P variant was found to reduce the K^+^ current amplitude [19]. Therefore, amino acid substitutions at A242 appear to greatly increase the risk of seizure susceptibility, likely by destabilizing the channel’s open state leading to impaired neuronal repolarization and increased neuronal excitability. 

### 4.3. A Frameshift Variant Affecting Both Voltage-Sensing and Pore Domains

A de novo heterozygous frameshift mutation P264LfsTer10 was identified in a female patient with EA1, episodes of generalized myoclonic seizure, mild cognitive impairment related to epileptic encephalopathy, asterixis (i.e., a sudden drop of the wrist or arm), falls, and cerebellar atrophy [33]. This is the first frameshift mutation discovered for *KCNA1*. The mutation introduces a premature stop codon that terminates the protein in the central portion of S3, thereby eliminating part of the voltage-sensing domain and the entire pore domain (Figure 1). Because of the severity of the truncation, this variant is predicted to be a null allele; however, the abbreviated protein could also exert dominant negative effects (see below). 

The P264LfsTer10 mutation is reminiscent of the megencephaly (*mceph*) mouse model of *Kcna1,* which has epilepsy due to an 11-nucleotide deletion causing a frameshift that truncates the Kv1.1 protein at amino acid 230 in the S2 domain [89,90,91,92]. The structural consequences of the human P264LfsTer10 mutation and the mouse *mceph* mutation are very similar. Both cause truncations that eliminate the critical domains needed for ion channel conductance and efficient voltage sensing, leaving intact only the first N-terminal half of the protein. Although both mutations cause epilepsy, the two alleles behave differently. In the patient, the mutation caused epilepsy by an autosomal-dominant mechanism, whereas in *mceph* mice, the mutation shows recessive inheritance requiring homozygosity for epilepsy manifestation [33,92]. 

Functional and expression studies are lacking for the human P264LfsTer10 variant, but experiments in *mceph* mice provide clues about the consequences of such severe truncation mutations. In the *mceph* mouse model, *Kcna1* mRNA is still present at high levels suggesting that the transcript avoids destruction through intrinsic cell protection mechanisms such as nonsense-mediated decay (NMD) [89,93]. This lack of degradation could be due to the lack of introns in *Kcna1,* which can render genes more resistant to NMD [93]. In functional studies using Xenopus oocytes, the *mceph*-encoded protein acts as a dominant-negative mutation when expressed with other Kv family subunits, such as Kv1.2 and Kv1.3 [89]. This dominant negative effect is hypothesized to result from the remaining N-terminus of the protein associating with other α subunits to form channels that become trapped in the endoplasmic reticulum (ER) and fail to reach the cell surface [89]. Supporting this idea, in Xenopus oocytes, the expression of MCEPH in equal ratios with either Kv1.2 or Kv1.3 reduced their expression at the plasma membrane [89]. Furthermore, Kv1.2 or Kv1.3 channels containing MCEPH that make it to the plasma membrane surface show reduced current and conductance [89]. Although there has not been extensive electrophysiological or protein analysis performed regarding the human P264LfsTer10 mutation, it is possible that these two mutations cause disease by similar mechanisms given their extremely similar structural consequences.

### 4.4. Evidence for the Possible Influence of Genetics Modifiers

#### 4.4.1. Findings in Patients

Recently described mutations in the Kv1.1 pore domain (G376S, G396R, and G396V) provide evidence for the possibility of genetic modifiers altering the clinical presentations of *KCNA1* channelopathy, especially with regard to epilepsy phenotypes. The G376S mutation was identified in male and female siblings who both exhibited seizures, typical EA1 symptoms, myokymia, and learning difficulties [29]. The mutation affects the S5–S6 extracellular linker, which composes part of the channel pore and provides the ion selectivity filter. Despite carrying the same mutation, the two siblings exhibited key differences in the severity of their seizure disorders and comorbidities. The older brother had an earlier seizure onset at 11 months of age compared to his younger sister who began having seizures at three years of age [29]. The older brother also had more severe tonic-clonic seizures, whereas the younger female sibling had seizures resembling absence spells [29]. Seizures in both siblings were often triggered by fever, exercise, fatigue, emotional stress, or illness [29]. Although both siblings had learning difficulties, the older brother had more pronounced cognitive impairment, developmental delay, and slurred speech, which were absent in the younger sister [29]. Therefore, the earlier seizure onset, more severe seizure-associated behaviors, and the presence of worse neurodevelopmental deficits in the older brother suggest he was more severely affected than his younger sister with the same mutation. Thus, this sibling pair demonstrates how clinical presentation can vary among patients, even those carrying the same mutation. One explanation for this variation could be the presence of genetic modifiers that alter genotype–phenotype correlations. In addition, sex differences could also play a role.

Variable epilepsy phenotypes are also observed for recently identified mutations at G396 and A242. A G396R variant affecting the S6 pore domain region of the protein was found in a patient with idiopathic generalized epilepsy, paroxysmal dyskinesia, and myokymia [52]. At approximately the age of 6 years, the patient was also diagnosed with cognitive disabilities including attention deficit hyperactivity disorder (ADHD) and mild difficulty in expressive language [52]. However, another new variant, G396V, was discovered at this same amino acid location in a different patient who was diagnosed with paroxysmal kinesigenic dyskinesia without the presence of epilepsy or seizures [52]. Differing disease phenotypes are also seen for two different missense mutations affecting A242. Whereas an A242S variant was found to cause EA1, myokymia, and epileptic encephalopathy (West Syndrome) as discussed previously above [20], another new variant (A242T) was identified in a patient presenting with myokymia but without epilepsy or seizures [21]. Additionally, variable phenotypes have also previously been described for other patients with different missense mutations affecting identical amino acid variants at A261, T226, V408, and F414 [1,10,14,15,16,17,18,28,29,58,59]. While the effects of environmental factors or the nature of the specific amino acid changes cannot be ruled out, we hypothesize that the presence of genetic modifiers is likely contributing at least partially to the differences in clinical presentation between patients, especially with regard to epilepsy.

#### 4.4.2. Findings in Mouse Models

Studies in mouse models provide support for the ability of genetic modifiers to significantly alter the clinical presentation of *KCNA1*-related channelopathy. At least six genes have been identified that can modify epilepsy phenotypes in the *Kcna1* global knockout (KO) mouse model including *Cacna1a* (a P/Q-type calcium channel α subunit gene) [94]; *Mapt* (a tau microtubule-binding protein gene) [95]; *Bad* (a BCL2-associated agonist of cell death gene) [96]; *Scn2a* (a Nav1.2 voltage-gated sodium channel gene) [97]; *Scn8a* (a Nav1.6 voltage-gated sodium channel gene) [98]; and *Slc7a11* (a System x-c glutamate antiporter gene) [99] (Table 3). These genetic modifiers usually exhibit a reduction in seizures and/or the incidence of sudden unexpected death in epilepsy (SUDEP) in *Kcna1* KO mice.

*Scn8a* and *Slc7a11* are the two most recently identified genes that can modify aspects of epilepsy in *Kcna1* KO mice. In a study exploring the efficacy of anti-sense oligonucleotide (ASO) therapy for the treatment of epilepsy, a reduction in *Scn8a* brain expression levels by ASO was found to extend the lifespan and delay SUDEP onset in *Kcna1* KO mice; however, seizure burden was not significantly improved [98]. In a second study, *Slc7a11*; *Kcna1* double KO animals were generated to investigate mechanisms of neurogenesis and epileptogenesis [99]. Genetic knockout of *Slc7a11* was found to have unique modifying effects in *Kcna1* KO mice, improving the megencephaly phenotype associated with *Kcna1* deletion but not significantly changing seizure severity or SUDEP incidence [99]. Thus, the *Slc7a11* mutation seems to have beneficial effects on aberrant postnatal neurogenesis in *Kcna1* KO mice without significantly altering epileptogenesis. These recent studies in mice add to the growing list of genetic modifiers that can significantly alter phenotypes associated with epilepsy due to *Kcna1* mutation. 

### 4.5. Potential Association between Epilepsy Variants and Respiratory Dysfunction

#### 4.5.1. Findings in Patients

Two new *KCNA1* variants (L296F and G396R) were recently discovered with links to epilepsy and breathing difficulties [37,52], suggesting a previously unrecognized connection between *KCNA1* channelopathy and respiratory phenotypes, especially in epilepsy patients. With the addition of these mutations, the total number of *KCNA1* variants linked to respiratory dysfunction now stands at six, which accounts for approximately 9% (6/65) of all pathogenic or likely pathogenic *KCNA1* mutations (Table 2). Notably, 83% (5/6) of the *KCNA1* variants associated with respiratory dysfunction occur in patients with epilepsy (Table 1). Three of the five respiratory-epilepsy *KCNA1* variants occur in the S6 pore domain, including missense mutations G396R and P403S, and a copy number variant (CNV) [52,53,55]. The CNV results in five extra copies of the distal portion of S6, which likely renders the protein non-functional [53]. The other two respiratory-epilepsy variants localize to S2 (T226R) and S4 (L296F) [17,18,37]. Finally, the lone variant that causes respiratory impairment in the absence of epilepsy is an in-frame three-nucleotide deletion causing loss of the phenylalanine at amino acid 250 (FF > F250) in the S2–S3 linker; this mutation is associated with EA1 [23,24].

The descriptions of breathing dysfunction in patients with *KCNA1* channelopathy raise concern that it could increase the risk of sudden unexpected death in epilepsy (SUDEP), the leading cause of epilepsy-related mortality [100,101,102]. When SUDEP has been witnessed in patients, it consistently involves the development of breathing abnormalities following a generalized tonic-clonic seizure, which progresses to terminal apnea and then cardiac arrest [100]. Three variants (L296F, G396R, and the CNV in S6) have been linked to breathing dysfunction specifically during seizures and/or the postictal period [37,52,53], which would be predicted to increase the risk of SUDEP. The patient with the L296F mutation had recurrent focal seizures accompanied by prolonged ictal/postictal oxygen desaturation and cyanosis [37], while the patient with the G396R mutation experienced generalized tonic-clonic seizures and had a febrile convulsive status epilepticus episode that required intubation for respiratory support [52]. The CNV patient exhibited hemiclonic and prolonged partial seizures that resulted in breathing cessation and cyanosis [53]. At the age of 3, the CNV patient was found cyanotic and unresponsive in bed, deceased from SUDEP [53]. However, it should be noted that the CNV patient also carried a mutation in the voltage-gated sodium channel gene *SCN1A*, which is strongly implicated in SUDEP; therefore, it is possible the phenotypes were dually influenced by both mutations [53].

Another important feature of SUDEP is that it tends to occur at night when patients are in bed and presumably asleep. Two *KCNA1* variants have been identified in epilepsy patients with breathing issues occurring specifically at night or during sleep. The P403 mutation was associated with very loud nighttime breathing in a patient [55], while the T226R variant was linked to sleep apnea, hypopnea, and hypoxemia [17,18].

#### 4.5.2. Findings in Mouse Models

An association between *KCNA1* mutations and aberrant respiratory function is supported by findings in *Kcna1* knockout (KO) mice. *Kcna1* KO mice are a commonly used model for investigating potential mechanisms underlying SUDEP pathophysiology. They exhibit many of the same features seen in humans, including generalized tonic-clonic seizures, seizure-induced sudden death, and ictal cardiorespiratory dysfunction [103,104,105,106,107]. During seizures, *Kcna1* KO mice display various abnormal breathing patterns, such as ataxic breathing, hypopnea, and apnea, which always precede cardiac abnormalities [104]. Consequently, respiratory dysfunction is thought to be a primary factor contributing to SUDEP risk in this model. In addition, KO mice exhibit changes in respiratory function during non-seizure periods, such as increases in breathing rate and respiratory variability, and reductions in sigh-apnea coupling and oxygen saturation [104,108]. 

Using a bioengineering systems approach to study inter-organ directed connectivity, *Kcna1* KO mice exhibit elevated brain-lung connectivity (compared to wildtype animals), which becomes further augmented during seizure periods [109]. This suggests aberrant basal neuro-respiratory communication that becomes more impaired by seizures. Immunohistochemistry experiments show the presence of Kv1.1 protein in brain regions controlling respiration [110], suggesting that Kv1.1-containing channels contribute to intrinsic control of breathing. In *Kcna1* KO mice, the absence of Kv1.1 in these brain networks results in extensive astrogliosis and microgliosis, indicating seizure-induced brain damage to these respiratory neurocircuits [110].

In summary, given that seizure-related respiratory failure is a suspected primary cause of SUDEP and that respiratory dysfunction is a shared feature of *KCNA1* channelopathy in both patients and mouse models, we propose that individuals with epilepsy due to *KCNA1* mutations should be assessed for respiratory performance to identify those at higher risk of SUDEP and to provide enhanced surveillance.

## 5. New Non-Epilepsy Related Variants

Of the newly discovered *KCNA1* variants, nine of the mutations are not related to epilepsy but are instead linked with other diseases such as EA1, myokymia, musculoskeletal abnormalities, and nystagmus. The association of several of these new variants with musculoskeletal and nystagmus phenotypes indicates that these two conditions may be more prevalent in *KCNA1* channelopathy than previously thought. Additionally, the new non-epilepsy-related variants include the first three pathogenic mutations ever found to impact the intracellular N-terminal portion of the protein. Prior to their discovery, the N-terminus and the S1–S2 linker were the only two domains of the protein for which pathogenic mutations had not been identified.

### 5.1. Episodic Ataxia Type 1 (EA1) Mutations

Most mutations in *KCNA1* cause EA1. Unlike the epilepsy-associated mutations, which predominate in the pore domain regions, EA1-associated variants are distributed relatively evenly across the whole length of the protein in all regions (Table 2, Figure 1). However, variants in S1 have the highest rate of association with EA1 with 100% (8/8) of mutations there causing the disease (Table 2). Out of the seventeen new *KCNA1* variants reviewed here, eight cause EA1 phenotypes. Perhaps unsurprisingly, these newly found mutations occur in nearly every domain of the protein, including in S1, which has been shown to have an especially strong association with EA1. The newly found variant in S1 is a V174A mutation, which is the same location as a different previously described EA1 mutation (V174F) [1,8,9]. Additionally, a patient was identified with EA1 due to an L155P mutation that is noteworthy because it is one of only three pathogenic variants ever found to affect the N-terminus of the protein [5]. The other two were also recently discovered and they are associated with musculoskeletal abnormalities, as discussed below.

### 5.2. Myokymia Mutations

Myokymia is a common manifestation of *KCNA1* channelopathy, occurring in 52% of pathogenic or likely pathogenic *KCNA1* variants and usually in combination with EA1 (82% of the time). The myokymia-associated *KCNA1* variants are present across most of the protein domains with the highest rates occurring in S2 (88%) and the S2–S3 linker (100%) regions. Out of the 17 recently identified *KCNA1* variants, eight display myokymia phenotypes, including two in the S2 domain affecting the same amino acid (A242T and A242S) [20,21]. The A242T variant was reported in a patient with symptoms of persistent limb myokymia including in the overnight hours during sleep [21]. The A242S mutation was found in a patient with a combination of myokymia, EA1, and epilepsy [20]. The A242 residue may represent a hotspot for myokymia mutations within the S2 domain, which already exhibits a very high association with myokymia risk [6,19].

### 5.3. Emerging Phenotypes Revealed by New Variants

#### 5.3.1. Musculoskeletal Abnormalities

Overall, 17% of *KCNA1* mutations are reported to cause musculoskeletal phenotypes and they almost exclusively occur in the N-terminal half of the protein, which composes the voltage-sensing domain (Table 2). This percentage was increased by the discovery of four new mutations (E49Q, R86Q, P264LfsTer10, and T268K) that exhibit various musculoskeletal abnormalities, suggesting these types of deficits may be a more common feature of *KCNA1* channelopathy than previously thought. The newly identified E49Q mutation was linked to calf hypertrophy [3]. This specific phenotype was also reported previously in patients with P244H, L305F, and F184C variants [12,19,40], suggesting a correlation between *KCNA1* mutations and lower limb musculature. Additionally, a patient with another previously described variant, P403S, was reported to have “ample muscle bulk” but the location of this excessive muscle was not specified [55]. In addition to muscle hypertrophy, the recently discovered R86Q mutation was associated with muscle stiffness and pain [4]. Muscle stiffness and pain can also be features of neuromyotonia, which has been described in a patient with an A242P mutation [6,19]. The T268K mutation was identified in a patient with multiple musculoskeletal abnormalities including tip-toe ambulation, kyphoscoliosis, lumbar hyperlordosis, and flat feet [34]. Finally, as discussed above, the P264LfsTer10 mutation was associated with asterixis in which the wrist or arm would suddenly drop [33]. 

The discovery of the E49Q and R86Q mutations, along with the EA1-related L155P variant, brings the number of mutations affecting the intracellular N-terminal portion of Kv1.1 to three. Prior to the identification of those three variants, that part of the protein was devoid of pathogenic mutations. The N-terminal domain has been hypothesized to be important for regulating subunit assembly [74]. Electrophysiological studies of the human L155P mutation showed that outward K^+^ currents are eliminated in homomeric mutant channels, demonstrating that they are non-functional [5]. Furthermore, channels composed of 50/50 combinations of wildtype and L155P subunits exhibit peak currents that are reduced by more than half and altered gating kinetics with several-fold faster inactivation [5]. Previously, the lack of pathogenic variants in the N-terminal region suggested that mutations there were likely benign (i.e., not disease-causing) and consequently never clinically identified. However, these new discoveries reveal that variants in this region are not as innocuous as initially thought.

#### 5.3.2. Nystagmus

Nystagmus is a rare feature of *KCNA1* channelopathy occurring in 6% (4/65) of pathogenic *KCNA1* variants. Nystagmus is a condition characterized by the involuntary, rhythmic movement of the eyes, which can affect vision, balance, and coordination [111]. Until recently, only two *KCNA1* variants had ever been linked to nystagmus, F184C and F303V [12,39]. However, two new variants, E325Q and R295C, have been identified, doubling the number of mutations associated with this disease to four. The E325Q mutation was found in a patient with EA1 who displayed downbeat nystagmus, which is characterized as a pathological upward gaze followed by a corrective downward gaze to the right of the visual field [46]. Another mutation, E325D, has been identified at this same amino acid residue but that patient exhibited EA1 without nystagmus [45]. The R295C mutation was discovered in a patient with nystagmus and cervical dystonia, which causes the neck muscles to contract involuntarily leading to painful head turns [36]. Two of the four nystagmus variants map to S4 but the small total number of *KCNA1* variants associated with nystagmus limits the reliability of drawing any conclusions about a potential location bias. However, as more patients are discovered with nystagmus due to *KCNA1* channelopathy, a more reliable correlation between the genotype and the phenotype may be revealed.

## 6. Summary and Conclusions

Recent research has revealed new pathogenic gene variants in *KCNA1* channelopathy, providing valuable insights into genotype–phenotype correlations. Mapping the distribution of variants across different protein domains has highlighted several important patterns. For example, epilepsy-causing mutations are most common in the S5–S6 pore domain, with the most severe forms of the disease associated with the PVP motif of S6. EA1 and myokymia, the most common diseases associated with *KCNA1* variants, show relatively even mutation distributions across the various protein domains. However, mutations in the S1 domain and in S2 and S2–S3 linker domains appear to have particularly high associations with EA1 and myokymia, respectively. The discovery of new variants located in the intracellular N-terminal domain has expanded our understanding of the protein regions associated with the disease. The only domain of the Kv1.1 protein for which no pathogenic mutations are yet known is the S1–S2 linker, but further research is needed to determine whether this absence is simply coincidental or due to mutations in this region being benign. Finally, the recently identified variants reveal new associations between *KCNA1* mutations and musculoskeletal disease and nystagmus, thus expanding the known phenotypic spectrum of *KCNA1* channelopathy.

The newly identified mutations have also expanded the functional and molecular nature of known variants. The first GOF mutations were recently found, revealing new therapeutic avenues for treating *KCNA1*-related diseases with pre-existing potassium channel blocking agents. Additionally, new potassium channel opener drugs are being developed that could treat the more traditional loss-of-function variants [112,113]. Among the recently discovered variants was also the first frameshift *KCNA1* mutation, which results in extensive protein truncation of the C-terminal half of the protein and shares significant similarities with the *mceph* mouse mutant, offering an excellent model to investigate the consequences of such a mutation. 

The discovery of new *KCNA1* mutations has provided significant insights into *KCNA1*-related epilepsy. Notably, the presence of siblings with the same mutation but differing phenotypes provides support for the role of genetic modifiers. Additionally, recent findings in mouse models have added to the growing list of genes that can modify *Kcna1*-related epilepsy. The discovery of several new *KCNA1* variants that cause respiratory dysfunction related to seizures and/or sleep is particularly relevant for SUDEP, suggesting that epilepsy patients with *KCNA1* mutations may be at increased risk of SUDEP and should receive enhanced surveillance. In summary, the new genetic discoveries in *KCNA1* channelopathy deepen our understanding of the relationship between genotype and phenotype, promising to improve the clinical management of patients through more accurate diagnosis, prognosis, and targeted therapeutic interventions.

## Figures and Tables

**Table 1:** Pathogenic or likely pathogenic human *KCNA1* mutations and their associated clinical phenotypes.

Mutation	Protein Domain	Clinical Diagnoses	Other Clinical Observations	Reference
**E49Q**	N Terminus	MSk		[3]
**R86Q**	N Terminus	MSk		[4]
**L155P**	N Terminus	EA1		[5]
R167M	S1	EA1 + Myokymia + MSk		[6]
A170S	S1	EA1		[7]
**V174A**	S1	EA1 + Myokymia		[8]
V174F	S1	EA1 + Myokymia		[1,9]
I176R	S1	EA1		[10]
I177N	S1	EA1		[11]
F184C	S1	EA1 + Seizures + Nystagmus + MSk + Myokymia		[12]
C185W	S1	EA1 + Hyperthermia + Myokymia	Sleep ^a^	[6,13,14]
T226A	S2	EA1		[10]
T226M	S2	EA1 + Myokymia		[15]
T226K	S2	Myokymia + MSk		[16]
T226R	S2	EA1 + Epilepsy + Myokymia + MSk	Respiratory ^b^, Sleep ^c^, DD	[17,18]
R239S	S2	EA1 + Myokymia		[1]
A242P	S2	Myokymia + Seizures		[6,19]
**A242S**	S2	EA1 + Myokymia + EE	DD	[20]
**A242T**	S2	Myokymia		[21]
P244H	S2–S3 IL	Myokymia + MSk		[19]
F249C	S2–S3 IL	EA1 + Myokymia + Hyperthermia		[22]
F249I	S2–S3 IL	EA1 + Myokymia		[1]
FF > F250	S2–S3 IL	EA1 + Myokymia	Respiratory ^d^	[23,24]
N255D	S3	Hypomagnesemia		[25,26]
N255K	S3	PKD		[27]
**A261T**	S3	EA1 + Myokymia + Seizures		[28,29]
I262T	S3	EA1		[30,31]
I262M	S3	EA1 + Myokymia	Mild ID	[32]
**P264LfsTer10**	S3	EA1 + EE + MSk		[33]
**T268K**	S3	Myokymia + MSk		[34]
E283K	S3–S4 EL	EA1 + Myokymia		[35]
**R295C**	S4	Nystagmus		[36]
**L296F**	S4	Epilepsy	Respiratory ^e^	[37]
V299I	S4	EA1 + PMC + Myokymia		[38]
F303V	S4	EA1 + Myokymia + Nystagmus		[39]
L305F	S4	EA1 + Myokymia + MSk		[40]
R307C	S4	EA1		[41]
G311D	S4–S5 IL	EA1 + Myokymia		[42]
G311S	S4–S5 IL	EA1		[43]
I314T	S4–S5 IL	EA1 + Myokymia		[17]
L319R	S4–S5 IL	PKD + Seizures		[27]
R324T	S5	EA1 + Epilepsy + Myokymia		[44]
E325D	S5	EA1 + Myokymia		[45]
**E325Q**	S5	EA1 + Nystagmus		[46]
L328V	S5	Hypomagnesemia		[47]
L329I	S5	EA1		[48]
**G336E**	S5	EA1 + Myokymia + Seizures		[49]
S342I	S5	EA1 + Seizures		[30,50]
V368L	S5–S6 pore loop	EE	Severe ID	[51]
**G376S**	S5–S6 pore loop	EA1 + Myokymia + Seizures	Moderate ID, DD	[29]
A395S	S6	EA1		*
**G396R**	S6	Myokymia + Epilepsy	Respiratory ^f^, ADHD, Mild ID	[52]
**G396V**	S6	PKD		[52]
CNV ^#^	PVP-S6	Epilepsy	Respiratory ^g^, Global DD	[53]
**P403A**	S6 (PVP)	Epilepsy	DD, ID	[54]
P403S	S6 (PVP)	EA1 + Epilepsy + Myokymia + MSk	Respiratory ^h^, DD, Moderate ID	[55]
V404I	S6 (PVP)	EA1 + Myokymia	Mild ID	[10,19,56]
P405S	S6 (PVP)	EE	DD, Macrocephaly ^i^	[55]
P405L	S6 (PVP)	EE	PDD ^j^	[55,57]
I407M	S6	EA1 + Myokymia		[6]
V408A	S6	EA1 + Myokymia		[1]
V408L	S6	EA1 + Seizures	Global DD	[58]
F414C	S6	EA1		[59]
F414S	S6	EA1 + Epilepsy		[14]
R417stop	C Terminus	EA1		[19]

Human SNP mutations were identified using the NCBI, ClinVar, and dbSNP databases. The full list of *KCNA1* mutations was filtered by the categories “Pathogenic” and “Likely Pathogenic.” The compiled list of human mutations was used as search criteria in PubMed to find clinical discussions of patients with these mutations and the functional research associated with them. Additional literature searches were also used to identify mutations not yet listed in the NCBI genetic databases. Recently identified mutations, which are the focus of this review, are shown as bolded and underlined. Myokymia may also include neuromyotonia as the two were frequently used interchangeably in the literature. Abbreviations: IL, intracellular linker; EL, extracellular linker; PVP, proline–valine–proline motif; MSk, musculoskeletal abnormalities; PKD, paroxysmal kinesigenic dyskinesia; EE, epileptic encephalopathy; PMC, paradoxical myotonic congenita; DD, developmental delay; ID, intellectual disability; PDD, pervasive developmental disorder; ADHD, attention-deficit/hyperactivity disorder. # Copy number variant (CNV) case resulting in 5 copies of the region from the PVP motif to the end of S6. * published citation could not be found; ClinVar variation label NM_000217.3(KCNA1):c.1183G > T (p.Ala395Ser) and accession number VCV000431378.; ^a^ self-reported needing only 5–6 h of sleep per night and being very active during the night; ^b^ recurrent apneic episodes with cyanosis; ^c^ prolonged sleep latency, reduced sleep efficiency, obstructive sleep apnea, hypopnea, ~80% oxygen desaturation during sleep; ^d^ difficulty breathing during attacks and isolated episodes of an inability to inhale; ^e^ ictal/postictal oxygen desaturation and cyanosis; ^f^ status epilepticus episode requiring intubation for respiratory support; ^g^ seizure at 4 months of age requiring cardiopulmonary resuscitation (CPR), seizures at 11 months were associated with cyanosis, found deceased at 3 years and 3 months of age cyanotic and unresponsive; ^h^ before age 2, very loud breathing at night; ^i^ head circumference in the 93rd percentile; ^j^ now also called autism spectrum disorder.

**Table 2:** Disease rates for pathogenic or likely pathogenic *KCNA1* variants in different protein domains.

Protein	No.	Disease or Symptom					
Domain	Mutations	EA1	Myokymia	Epilepsy	MSk	Respiration	Nystagmus
N	3	33%	0%	0%	67%	0%	0%
S1	8	100%	63%	13%	25%	0%	13%
S1–S2	0	0%	0%	0%	0%	0%	0%
S2	8	63%	88%	38%	25%	13%	0%
S2–S3	4	75%	100%	0%	25%	25%	0%
S3	7	57%	43%	29%	29%	0%	0%
S3–S4	1	100%	100%	0%	0%	0%	0%
S4	6	67%	50%	17%	17%	17%	33%
S4–S5	4	75%	50%	25%	0%	0%	0%
S5	7	86%	43%	43%	0%	0%	14%
S5–S6	2	50%	50%	100%	0%	0%	0%
S6	14	57%	36%	57%	7%	21%	0%
C	1	100%	0%	0%	0%	0%	0%
Total	65	69%	52%	32%	17%	9%	6%

The values shown represent the percentage of mutations in each Kv1.1 protein domain associated with the listed disease or phenotype. The individual cells of the table are color-coded in a heat map where white is the lowest value, and the darkest shade of blue is the highest. Percentages were calculated by dividing the number of mutations associated with the listed disease or symptom in the designated domain by the total number of mutations in that domain. Abbreviations: EA1, episodic ataxia type 1; MSk, musculoskeletal abnormalities.

**Table 3:** Genetic modifiers of *Kcna1* knockout mouse phenotypes.

Gene	Gene Function	Mutation Type	Impact on *Kcna1* Knockout Mouse Model	Ref.
*Cacna1a*	Calcium channel	Missense (*tottering* allele)	Reduced seizure frequency, increased survival	[94]
*Mapt*	MT associated protein	Gene knockout	Reduced seizure frequency, increased survival	[95]
*Bad*	Apoptosis	Gene knockout	Reduced seizure frequency, increased survival	[96]
*Scn2a*	Sodium channel	Gene knockout	Reduced seizure frequency, increased survival, improved brain-heart dynamics	[97]
*Slc7a11*	Glutamate antiporter	Gene knockout	Restored normocephalic brain	[99]
*Scn8a*	Sodium channel	ASO knockdown	Improved survival	[98]

Genetic modifiers of *Kcna1* knockout mouse phenotypes were identified through PubMed literature searches. Abbreviations: MT, microtubule; ASO, antisense oligonucleotide.

## Data Availability

Not applicable.
